# NAMPT and BMAL1 Are Independently Involved in the Palmitate-Mediated Induction of Neuroinflammation in Hypothalamic Neurons

**DOI:** 10.3389/fendo.2020.00351

**Published:** 2020-06-12

**Authors:** Andy Tran, Wenyuan He, Nan Jiang, Jim T. C. Chen, Denise D. Belsham

**Affiliations:** ^1^Department of Physiology, University of Toronto, Toronto, ON, Canada; ^2^Department of Medicine, University of Toronto, Toronto, ON, Canada; ^3^Department of Obstetrics and Gynaecology, University of Toronto, Toronto, ON, Canada

**Keywords:** obesity, NAMPT, visfatin, circadian, palmitate, hypothalamus, inflammation

## Abstract

Obesity is a prominent metabolic disease that predisposes individuals to multiple comorbidities, including type 2 diabetes mellitus, cardiovascular diseases, and cancer. Elevated circulating levels of fatty acids contribute to the development of obesity, in part, by targeting the hypothalamus. Palmitate, the most abundant circulating saturated fatty acid, has been demonstrated to dysregulate NAMPT and circadian clock proteins, as well as induce neuroinflammation. These effects ultimately result in hypothalamic dysregulation of feeding behavior and energy homeostasis. NAMPT is the rate-limiting enzyme of the NAD+ salvage pathway and its expression is under the control of the circadian clock. NAD+ produced from NAMPT can modulate the circadian clock, demonstrating bidirectional interactions between circadian and metabolic pathways. Using NPY/AgRP-expressing mHypoE-46 neurons as well as the novel mHypoA-BMAL1-WT/F and mHypoA-BMAL1-KO/F cell lines, we studied whether there were any interactions between NAMPT and the core circadian clock protein BMAL1 in the palmitate-mediated induction of neuroinflammation. We report that palmitate altered *Nampt, Bmal1, Per2* and the inflammatory genes *Nf-*κ*b, I*κ*B*α*, Il-6*, and *Tlr4*. Contrary to studies performed with peripheral tissues, the palmitate-mediated induction in *Nampt* was independent of BMAL1, and basal *Nampt* levels did not appear to exhibit rhythmic expression. Palmitate-induced downregulation of *Bmal1* and *Per2* was independent of NAMPT. However, NAMPT and BMAL1 were both involved in the regulation of *Nf-*κ*b, I*κ*B*α*, Il-6*, and *Tlr4*, as NAMPT inhibition resulted in the repression of basal *Nf-*κ*b* and *I*κ*B*α and normalized palmitate-mediated increases in *Il-6*, and *Tlr4*. On the other hand, BMAL1 deletion repressed basal *Nf-*κ*b*, but increased basal *Il-6*. We conclude that NAMPT and BMAL1 do not interact at the transcriptional level in hypothalamic neurons, but are independently involved in the expression of inflammatory genes.

## Introduction

Obesity predisposes individuals to comorbidities, such as type 2 diabetes mellitus (T2DM), cardiovascular diseases, and cancer (1). Increased consumption of fat is a main contributing factor to obesity (2), leading to elevated levels of saturated fatty acids (SFA) both in serum (3) and the hypothalamus (4). The arcuate nucleus of the hypothalamus contains critical orexigenic neuropeptide Y/agouti-related peptide (NPY/AgRP) and anorexigenic proopiomelanocortin (POMC) neurons. These neurons sense nutrients, such as fatty acids and peripheral hormones as signals of energy levels and release their respective neuropeptides to regulate energy homeostasis. Elevated SFA in the hypothalamus results in deleterious effects marked by the induction of neuroinflammation, oxidative stress, endoplasmic reticulum (ER) stress, and circadian disruption, all of which can lead to hormonal resistance and neuropeptide dysregulation (5–12).

Cells of the body, including hypothalamic neurons, possess an intrinsic clock machinery that generates a ~24-h circadian rhythm. Several core proteins are involved in the circadian machinery: circadian locomotor output cycles kaput (CLOCK) and brain and muscle ARNT-like 1 (BMAL1) are transcription factors that heterodimerize to bind to E-box elements, inducing the expression of genes encoding period (PER) and cryptochrome (CRY). PER and CRY are transcription factors that heterodimerize to repress CLOCK and BMAL1 expression, completing a transcriptional-translational feedback loop. Animal studies have shown that shift work induces circadian dysregulation and is associated with an increased risk for weight gain and obesity (13–15). Similar effects were also observed in shift workers (16, 17). Additionally, saturated fats such as palmitate can alter the expression and rhythmicity of BMAL1, CLOCK, and PER2 to alter feeding peptide expression (6).

NAD^+^ is involved in intracellular redox reactions and is a cofactor for enzymes, such as the deacetylase Sirtuin 1 (SIRT1) and the DNA repair poly enzyme poly ADP-ribose polymerase (PARP). These enzymes consume NAD+ to generate nicotinamide (NAM) as a by-product. The multistep conversion of NAM back to NAD^+^ is regulated by the rate-limiting enzyme nicotinamide phosphoribosyltransferase (NAMPT), also known as pre-B cell enhancing factor (PBEF) or visfatin. Importantly, the 5′ regulatory region of the *Nampt* gene contains E-box elements, which bind BMAL1:CLOCK heterodimers to drive expression. Critically, NAMPT mRNA and protein expression is decreased in the adipose tissue of HFD-fed mice (18, 19). Moreover, NAMPT expression is modulated by palmitate in the heart and the liver (20).

The hypothalamus is susceptible to developing neuroinflammation following the onset of HFD-feeding that precedes peripheral inflammation and weight gain (10, 21, 22). Hypothalamic neuroinflammation involves the secretion of inflammatory cytokines and reactive gliosis (9–11, 23). Critically, inflammation contributes to the disruption of energy homeostasis by altering feeding neuropeptide expression and the development of resistance to key anorexigenic hormones, such as leptin and insulin (24, 25). Many processes are implicated in the development of inflammation, such as the activation of toll-like receptor 4 (TLR4) or the intracellular metabolism of fats, both of which result in the activation of inflammatory NF-κB and MAPK signaling. NAMPT and BMAL1 may play also role in palmitate-induced inflammation as SIRT1 can target and modulate NF-κB activity (26), while BMAL1 has been demonstrated regulate expression of the inflammatory cytokine interleukin 6 (*Il-6*) (27, 28). However, whether the interaction between NAMPT or BMAL1 is involved in SFA-induced hypothalamic neuroinflammation remains unclear.

In this study, we investigated (1) whether the saturated fatty acid palmitate alters *Nampt, Bmal1*, and *Per2* expression in hypothalamic neurons, (2) the temporal sequence and mediators between the palmitate-induced modulations of *Nampt, Bmal1*, and *Per2* mRNAs, and (3) whether palmitate-induced neuroinflammation is mediated by either NAMPT or BMAL1. We used primary hypothalamic cultures and immortalized hypothalamic cell lines, including the NPY/AgRP-expressing clonal mHypoE-46 cell line, as well as the novel heterogenous mHypoA-BMAL1-WT/F and mHypoA-BMAL1-KO/F cell lines. We report that palmitate upregulates *Nampt* in hypothalamic neurons in contrary to reports in peripheral tissues. Both basal *Nampt* expression and the induction of *Nampt* by palmitate appear to be BMAL1-independent, and palmitate-mediated changes to *Bmal1* and *Per2* expression are independent of NAMPT. Thus, both NAMPT and BMAL1 appear to be independently involved in alterations to inflammatory *Nf-*κ*b, I*κ*B*α*, Il-6*, and *Tlr4* gene expression in response to palmitate.

## Methods

### Cell Culture and Reagents

Clonal mHypoE-46, and the heterogenous mHypoA-BMAL1-WT/F and mHypoA-BMAL1-KO/F cell lines were generated in our laboratory as previously described (29, 30). The mHypoA-BMAL1-KO/F cell line has been validated to lack BMAL1 protein, *Bmal1* mRNA, and clock rhythmicity (30). Cells were grown in low glucose Dulbecco's Modified Eagle Medium (DMEM) (MilliporeSigma; Etobicoke, ON, CA) containing 5.5 mM glucose, 2% fetal bovine serum (FBS) (Gibco, Burlington, ON, CA) and 1% penicillin-streptomycin (Gibco).

For primary culture experiments, whole hypothalamii from 8-week old male or female CD-1 mice (Charles River Laboratories, Senneville, QC, Canada) were extracted. Hypothalamii were gently triturated to achieve dispersion and cultured in 6-well plates for 7–9 days in neurobasal A medium (Gibco), supplemented with 10% FBS, 5% horse serum (Gibco), 1% PS, 1 × B27 serum-free supplement (Gibco) and 1 × GlutaMAX supplement (Gibco). 10 ng/μL CNTF was added to each well every day. After 7–9 days, cells were treated with 50 μM palmitate (MilliporeSigma) in low glucose DMEM (MilliporeSigma).

All animal procedures were approved by and performed in accordance with the Animal Use Protocols at the University of Toronto.

### *In vitro* Experimental Protocols

Sodium palmitate and FK866 were purchased from MilliporeSigma. Sodium palmitate was dissolved in molecular grade water by heating to 70°C to obtain a concentration of 50 mM. FK866 was dissolved in dimethyl sulfoxide (DMSO) (Thermo Scientific) to 10 μM. Treatments were diluted 1:1,000 in low glucose DMEM to reach the final concentrations as indicated in the results section. Cells were grown to to 75–80% confluency on 60 mm plates prior to treatment.

For time course experiments, mHypoA-BMAL1-WT/F and mHypoA-BMAL1-KO/F were grown to 75–80% confluency on 60 mm plates, serum-starved for 12 h and synchronized for 30 min with 20 μM forskolin (Thermo Scientific) in DMEM containing 5% FBS. This was then replaced with fresh DMEM containing 5% FBS, indicating *t* = 0 h. Cells were first harvested at 6 h, then afterwards at every 6-h interval up to 36 h. The samples used to assess *Nampt* rhythmicity were the same as those used in the study by Clemenzi et al. (31). With these studies, circadian oscillations of the core clock genes were demonstrated in the mHypoA-BMAL1-WT/F cells, and these rhythms were completely absent in the mHypoA-BMAL1-KO/F cell line.

### Quantitative Real-Time PCR (qRT-PCR)

Total mRNA was obtained using the PureLink RNA Mini Kit with on-column PureLink DNase (Ambion; Streetsville, ON, CAN) and quantified using the Nanodrop 2000c spectrophotometer. 1 μg of harvested RNA was used for cDNA synthesis with the High-Capacity cDNA Reverse Transcription Kit (Applied Biosystems). qRT-PCR was performed using gene specific primers (Table 1) and qRT-PCR was performed using gene specific primers and the Platinum SYBR Green qPCR SuperMix-UDG with ROX (Applied Biosystems) with the Applied Biosystems 7900 HT Real-Time PCR machine, then analyzed using SDS 2.4 software (Applied Biosystems). Relative mRNA quantities were determined using the ΔΔCt method or with the standard curve method and normalized to the reference gene *Rpl7*.

**Table 1:** List of primers used for qRT-PCR.

**Gene**	**Primer sequence (5^**′**^-3^**′**^)**	**Amplicon Size (bp)**
*Rpl7*	F: TCGCAGAGTTGAAGGTGAAG R: GCCTGTACTCCTTGTGATAGTG	114
*Nampt*	F: CTCCTTCAAGTGCAGCTATGT R: CCGCTGGTGTCCTATGTAAAG	122
*Bmal1*	F: GGGAGGCCCACAGTCAGATT R: GTACCAAAGAAGCCAATTCATCAA	78
*Nf*-κ*b*	F: AGTGACAGCGACAGTGACAACAGA R: TCATCAGGAAGAGGTTTGGCTGCT	119
*Iκbα*	F: TGCCTGGCCAGTGTAGCAGTCTT R: CAAAGTCACCAAGTGCTCCACGAT	150
*Il-6*	F: GTGGCTAAGGACCAAGACCA R: GGTTTGCCGAGTAGACTTCA	85
*Tlr4*	F: GCTTGAATCCCTGCATAG R: GCTCAGATCTAGGTTCTTGG	113

### Statistical Analysis

Results were analyzed using GraphPad Prism Software 6.0 (GraphPad Software; La Jolla, CA, USA) and presented as mean ± S.E.M. Data from each figure were analyzed using *t*-test or two-way ANOVA, followed by the Tukey or Sidak *post hoc* test. Circadian rhythmicity was determined using JTK Cycle to analyze the fit to a 24 h period (32). Statistical significance is denoted as ^*^P < 0.05, ^**^P < 0.01, ^***^P < 0.001, ^****^P < 0.0001.

## Results

### Palmitate Alters *Nampt, Bmal1*, and *Per2* mRNA Expression in Hypothalamic Neurons

**Figure 1:**
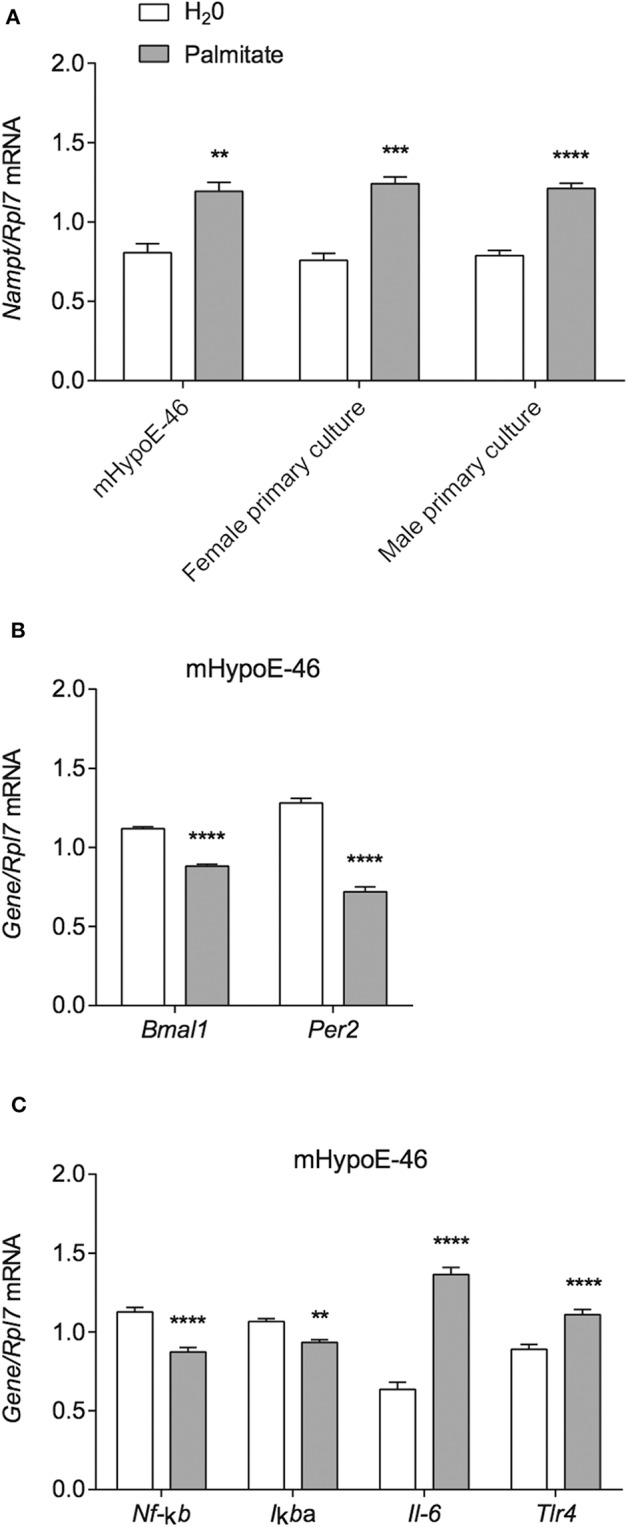
Palmitate alters expression of *Nampt*, core circadian clock genes, and inflammatory genes. mHypoE-46 neurons, as well as primary hypothalamic cultures derived from male and female CD-1 mice were treated with 50 μM palmitate for 24 h. Changes in mRNA expression of **(A)** *Nampt*, **(B)** *Bmal1* and *Per2*, or **(C)** *Nf-*κ*b, I*κ*B*α*, Il-6*, and *Tlr4* were assessed by qRT-PCR. *n* = 4 independent experiments. Data are expressed as mean ± SEM; **P < 0.01, ***P < 0.001, ****P < 0.0001 Statistical analysis was performed with an unpaired *t*-test.

Due to reports of altered *Nampt* mRNA in the periphery of DIO animals (18, 33), we investigated whether the same held true for hypothalamic neurons. Thus, we treated the immortalized mHypoE-46 cell line with 50 μM palmitate for 24 h and observed that palmitate upregulated *Nampt* (Figure 1A). We further validated our results in primary hypothalamic cultures derived from male and female CD-1 mice, observing a similar induction of *Nampt* with 50 μM palmitate treatment for 24 h (Figure 1A). Next, as both HFD and palmitate treatment have been shown to alter clock gene expression (6, 34), we were interested whether palmitate would also alter clock gene expression in mHypoE-46 neurons at the same time as *Nampt*. We observed that a 24 h treatment with 50 μM palmitate repressed both *Bmal1* and *Per2* mRNA (Figure 1B). As palmitate also altered inflammatory gene expression in hypothalamic neurons (10, 11), we then validated these findings in the mHypoE-46 model. Palmitate repressed *Nf-*κ*b* and *I*κ*b*α while elevating *Il-6* and *Tlr4* mRNA (Figure 1C).

### *Nampt* mRNA Expression Is Not Under the Control of BMAL1

**Figure 2:**
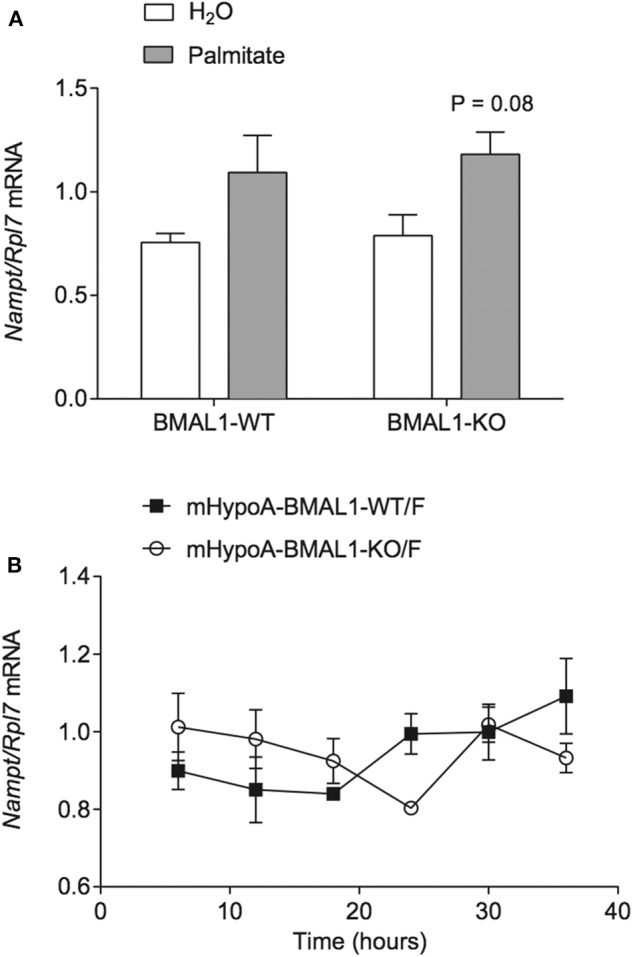
BMAL1 does not mediate the induction of *Nampt* by palmitate nor rhythmic NAMPT expression. **(A)** mHypoA-BMAL1-WT/F and mHypoA-BMAL1-KO/F cells were treated with 50 μM palmitate for 24 h and assessed for changes in *Nampt* mRNA. **(B)** mHypoA-BMAL1-WT/F and mHypoA-BMAL1-WT/F-BMAL1-KO/F cells were serum-starved for 12 h and synchronized for 30 min with 20 μM forskolin in DMEM containing 5% FBS. Fresh media containing 5% FBS was then applied, indicating t = 0 h. Cells were first harvested at 6 h, then every 6 h until 36 h. *Nampt* mRNA levels were assessed by qRT-PCR. *n* = 4 independent experiments. Data are expressed as mean ± SEM. Statistical analysis was performed by two-way ANOVA with the Tukey *post hoc* test.

Palmitate was previously shown to alter the expression of the core molecular clock genes *Bmal1, Clock*, and *Per2* in hypothalamic neurons over a 36 h time course (6), and interactions had been shown between core clock proteins and NAMPT expression. In mouse liver, *Nampt* mRNA and protein are rhythmically expressed (35). Furthermore, *Nampt* mRNA also demonstrated rhythmic expression in mouse embryonic fibroblasts (MEF), and the maintenance of *Nampt* expression required CLOCK as it was abolished in MEF-specific CLOCK knockouts (36). Accordingly, we sought to investigate the involvement of BMAL1 in palmitate-mediated induction of *Nampt*. We used the heterogeneous mHypoA-BMAL1-KO/F and mHypoA-BMAL1-WT/F cell lines, derived from female BMAL1-KO mice and BMAL1-WT female littermates, respectively. Using the same treatment paradigm as previously described, we observed that *Nampt* was not significantly induced by 24-h treatment of 50 μM palmitate in both mHypoA-BMAL1-KO/F and mHypoA-BMAL1-WT/F cell lines, although there was a trend toward increased expression in both cell lines (Figure 2A). We then questioned whether BMAL1 was required to impart rhythmic *Nampt* expression in hypothalamic neurons. mHypoA-BMAL1-KO/F and mHypoA-BMAL1-WT/F cell lines were serum-starved for 12 h then synchronized with 20 μM forskolin for 30 min, after which fresh DMEM was applied indicating *t* = 0 h. *Nampt* mRNA levels were not rhythmic over 24 h in mHypoA-BMAL1-WT/F (*P* = 0.41) nor mHypoA-BMAL1-KO/F cells (*P* = 1), and furthermore, basal *Nampt* mRNA levels were not significantly different between the two cell lines at any time point (Figure 2B). This was despite our previous finding that mHypoA-BMAL1-WT/F, but not mHypoA-BMAL1-KO/F cells, demonstrated rhythmic expression of *Bmal1, Per2, Agrp*, and *Pomc* mRNA (31).

### Intracellular NAMPT Activity Does Not Mediate the Changes in *Bmal1* and *Per2* mRNA by Palmitate

**Figure 3:**
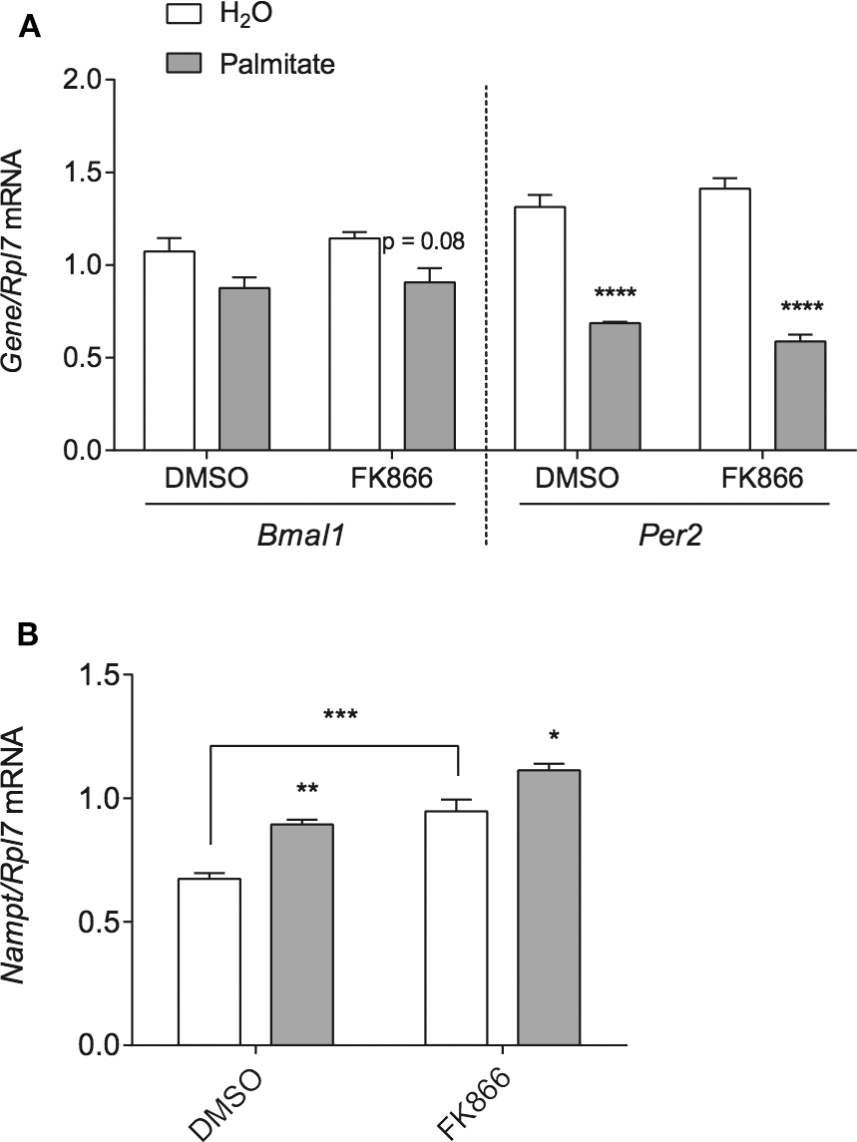
NAMPT activity does not mediate the repression of *Bmal1* nor *Per2* mRNA by palmitate. mHypoE-46 neurons were pretreated for 1 h with 10 nM FK866 or DMSO vehicle control, then treated with 50 μM palmitate for 24 h. Changes in **(A)** *Bmal1* and *Per2* mRNA or **(B)** *Nampt* mRNA were assessed by qRT-PCR. *n* = 4 independent experiments. Data are expressed as mean ± SEM, *P < 0.05, **P < 0.01, ***P < 0.001, ****P < 0.0001. Statistical analysis was performed by two-way ANOVA with the Tukey *post hoc* test.

Since NAMPT is able to regulate the circadian clock by the targeting BMAL1:CLOCK via NAD+ and SIRT1 (6, 35–40), we hypothesized that increased *Nampt* mRNA expression and thereby NAMPT and NAD+ production, would be responsible for the repression of *Bmal1* and *Per2* by palmitate. To block any potential increase in NAMPT enzymatic activity associated with the palmitate-mediated induction of *Nampt* mRNA, we treated mHypoE-46 neurons with 10 nM of specific NAMPT inhibitor inhibitor FK866. 24- and 48-h treatment with FK866 was effective at lowering NAMPT activity and NAD+ levels in hepatocarcinoma cells, hepatocytes, and neuroblastoma cells. We pre-treated with 10 nM FK866 for 1 h, followed by 24-h treatment with 50 μM palmitate. However, palmitate was unable to significantly repress *Bmal1* mRNA in the presence of DMSO (as opposed to Figure 1B where DMSO was not present). Moreover, FK866 failed to attenuate the repression of *Bmal1* and *Per2* by palmitate (Figure 3A). As a positive control, we observed that FK866 elevated basal *Nampt* mRNA expression, but it failed to normalize the increase of *Nampt* by palmitate (Figure 3B).

### NAMPT and BMAL1 Are Involved in Inflammatory Gene Expression

**Figure 4:**
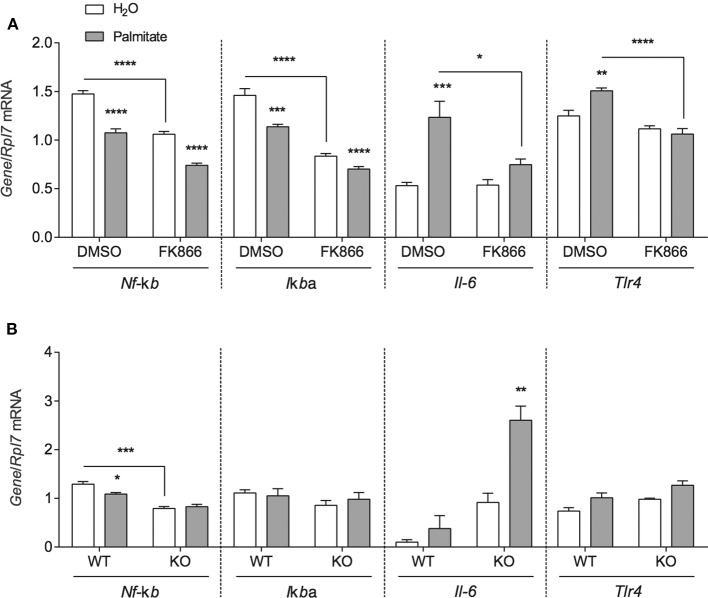
NAMPT and BMAL1 are involved in the induction of inflammatory gene expression by palmitate. **(A)** mHypoE-46 neurons were pretreated for 1 h with 10 nM FK866 or DMSO vehicle control then treated with 50 μM palmitate for 24 h. **(B)** mHypoA-BMAL1-WT/F and mHypoA-BMAL1-KO/F cells were treated with 50 μM palmitate for 24 h. Changes in *Nf-*κ*b, I*κ*B*α*, Il-6*, and *Tlr4* were assessed by qRT-PCR. *n* = 3–4 independent experiments. Data are expressed as mean ± SEM, *P < 0.05, **P < 0.01, ***P < 0.001, ****P < 0.0001. Statistical analysis was performed by two-way ANOVA with the Tukey *post hoc* test.

To investigate whether the inflammatory effects of palmitate could be attributed to altered NAMPT levels and activity, mHypoE-46 cells pre-treated with 10 nM FK866 for 1 h followed by 50 μM palmitate for 24 h and were assessed for changes in inflammatory *Nf-*κ*b, I*κ*B*α*, Il-6*, and *Tlr4* mRNA. In the presence of 10 nM FK866, basal *Nf-*κ*b* and *I*κ*B*α expression were repressed, but the NAMPT inhibitor had no effect on the palmitate-mediated downregulation of these genes (Figure 4A). The induction of *Il-6* and *Tlr4* mRNA were blocked by 10 nM FK866 pre-treatment (Figure 4A).

Due to reports that IL-6 production and secretion were regulated by BMAL1 in macrophages and microglia (27, 28), we were interested whether BMAL1 also plays a role in mediating the effects of palmitate on *Nf-*κ*b, I*κ*b*α *Il*-6, and *Tlr4*. mHypoA-BMAL1-KO/F and mHypoA-BMAL1-WT/F cells were treated with 50 μM palmitate for 24 h. We observed a significant induction in *Il-6* mRNA in mHypoA-BMAL1-KO/F cells with palmitate, but not in mHypoA-BMAL1-WT/F cells, suggesting an inhibitory role of BMAL1 on *Il-6* expression (Figure 4A). Similar to mHypoE-46 neurons pre-treated with 10 nM FK866, mHypoA-BMAL1-KO/F cells expressed lower basal levels of *Nf-*κ*b* than in mHypoA-BMAL1-WT/F cells (Figure 4B). Additionally, palmitate repressed *Nf-*κ*b* mRNA in mHypoA-BMAL1-WT/F cells, but not mHypoA-BMAL1-KO/F cells (Figure 4B). No significant changes were observed for *I*κ*b*α nor *Tlr4* (Figure 4B).

## Discussion

The underlying mechanisms for the pathogenesis of obesity are multifaceted and complex. Disruption of hypothalamic function by elevated saturated fatty acids is implicated in this disease as it affects peptidergic circuits that regulate feeding behavior and whole-body energy homeostasis. Importantly, the ability of saturated fats to alter NAMPT and the circadian clock as well as induce neuroinflammation has been suggested to contribute to hypothalamic dysfunction. However, it is unknown whether these processes are coupled in the hypothalamus. We demonstrate that palmitate-induced alterations of the circadian clock genes *Bmal1* and *Per2* as well as *Nampt* occur independently of each other in contrast to what had been observed in peripheral tissues. Despite this, our results suggest a role for NAMPT and BMAL1 in regulating inflammatory gene expression in the hypothalamus.

We report that treatment with the saturated fatty acid palmitate upregulated *Nampt* mRNA expresion in the mHypoE-46 hypothalamic cell line as well as in male-derived and female-derived primary hypothalamic cultures. A number of studies (18, 33, 41) found that *Nampt* mRNA and NAMPT protein levels were decreased in the liver of DIO mice. Another study similarly found a decrease in *Nampt* mRNA and protein expression in adipose tissue in DIO mice (18). A similar downregulation in hypothalamic NAMPT mRNA and protein was also observed following 6 and 8 weeks of HFD-feeding (42). Although the precise molecular mechanisms behind changes in NAMPT expression remain poorly understood, some studies suggest an inhibitory role of inflammatory cytokines on *Nampt* expression in the periphery. The cytokines IL-6 and tumor necrosis factor alpha (TNF-α) were demonstrated to repress *Nampt* expression in the adipose tissue of obese mice (43, 44). Similarly, *in vitro* studies have also demonstrated the repression of *Nampt* mRNA in adipocytes when stimulated by IL-6 and TNF-α (43, 45, 46). In contrast to a downregulation of *Nampt* in peripheral tissues, we instead report an induction of hypothalamic *Nampt* by palmitate. As palmitate is known to induce inflammatory signaling, the induction of *Nampt* may be driven by activation of the inflammatory transcription factors NF-κB and AP-1 as the respective response elements have been described in the *Nampt* 5' regulatory region (47, 48). The difference between the acute induction of hypothalamic *Nampt* in our study vs. downregulation in the hypothalamus following chronic consumption of high fat may be attributed to the temporal dynamics of the neuroinflammatory response. Previous studies have shown an induction of inflammatory markers in the hypothalamus within 24 h of exposure to high fat which temporarily subsides in the following days and weeks (10, 21). However, continued consumption of high fat ensures the return of inflammation to the hypothalamus.

The palmitate-mediated upregulation of *Nampt* mRNA may also result from the induction of oxidative stress. When cells are exposed to palmitate, reactive oxygen species (ROS) are generated and can damage DNA (49). DNA repair requires the NAD+ dependent enzyme PARP, which is a DNA nick sensor (49, 50). Hyper-activation of PARP following ROS-induced DNA damage can rapidly deplete intracellular NAD+, and the subsequent upregulation of *Nampt* expression could be a response to restore NAD^+^ levels similar to the *Nampt* induction observed with FK866.

Unlike reports in peripheral tissues, our study demonstrates that *Nampt* mRNA expression does not appear to be rhythmic in hypothalamic neurons. Furthermore, the expression of *Nampt* mRNA did not appear to require BMAL1 as expression levels were not significantly different between the mHypoA-BMAL1-WT/F and mHypoA-BMAL1-KO/F cell lines. This is in contrast to a study that reported abolished *Nampt* oscillation in the livers of CLOCK mutant mice (36). Similarly, in a rodent shift-work model that forces activity during the normal resting period, the expression of *Nampt* was decoupled from circadian clock in the liver, resulting in a loss of rhythmicity (51). Although we did not observe rhythmic *Nampt* expression, *Agrp, Pomc, Bmal1*, and *Per2* possessed circadian rhythmicity in the same mHypoA-BMAL1-WT/F cells, demonstrating that this cell line possesses a functional circadian clock (31). While this would indicate that *Nampt* does not appear to have rhythmic expression in hypothalamic neurons, we would suggest some caution with this interpretation as these results are from a set of heterogeneous cell lines. Further experiments will be undertaken using clonal cell lines that have previously been used in the analysis of circadian regulation of neuropeptide expression.

Furthermore, palmitate failed to increase *Nampt* in mHypoA-BMAL1-WT/F and mHypoA-BMAL1-KO/F cells. However, trends toward increased *Nampt* were detected in both cell lines with palmitate, suggesting that BMAL1 might not regulate *Nampt* expression in this model. As we also observed a repression in *Bmal1* and *Per2* mRNA with palmitate in mHypoE-46 neurons, this suggests overall repression of core circadian clock components, making it unlikely to be involved in the induction of *Nampt*. A limitation to this interpretation is that it is unknown whether the observed repression is due to changes in the phase or amplitudes of rhythmic *Bmal1* and *Per2* expression. We have previously reported significant reductions in the amplitudes, but not the phases, of *Bmal1* and *Per2* mRNA in a similar NPY/AgRP-expressing mHypoE-44 model (6). However, it is still possible that palmitate alters the rhythmic properties of clock genes in mHypoE-46 neurons differently than in mHypoE-44 neurons.

Neuroinflammation is characteristic of obesity and a consequence of HFD-feeding. Specifically, NF-κB signaling is a key contributor to the deleterious effects of palmitate. For example, NF-κB inhibition was able to diminish the palmitate-mediated increases in *Npy* and inflammatory gene expression (10, 12, 52). Our results demonstrate that NAMPT inhibition with FK866 had an anti-inflammatory effect, ablating the induction of *Il-6* and *Tlr4* mRNA by palmitate. TLR4 plays an important role in the induction of neuroinflammation by palmitate and signaling downstream of the receptor leads to NF-κB activation via degradation of the inhibitory IκBα subunit. This results in the translocation of NF-κB into the nucleus where it drives the expression of inflammatory genes including *Il-6* (53). Furthermore, FK866 also downregulated basal *I*κ*b*α and *Nf-*κ*b* mRNA, but did not normalize the palmitate-mediated downregulation of these genes suggesting that FK866 and palmitate repress *Nf-*κ*b* and *I*κ*b*α expression via independent mechanisms. The FK866-mediated repression of basal *Nf-*κ*b* and *I*κ*b*α may lead to lower protein levels, thus decreasing the ability of palmitate to drive *Il-6* and *Tlr4* expression through NF-κB signaling (53–56). Together, these findings suggest that FK866 may prevent the induction of inflammatory genes by targeting NF-κB at the mRNA or protein level.

Besides NAMPT, the circadian clock is an established regulator of immune responses in microglia and macrophages (27, 57). Studies have shown that BMAL1 regulates *Il-6* expression and secretion (27, 28). Specifically, BMAL1 in monocytes was involved in negatively regulating circulating levels of inflammatory cytokines including CCL2, IL-1β, IL-6 and IFN-γ, and furthermore, BMAL1 deletion resulted in higher expression of *Ccl2* and *Ccl8* mRNA in monocytes and macrophages (58). Moreover, circadian dysregulation or low BMAL1 expression resulted in higher circulating levels of pro-inflammatory cytokines (59). In agreement with these studies, we saw that *Il-6* expression was higher in mHypoA-BMAL1-KO/F cells than in mHypoA-BMAL1-WT/F cells, suggesting a negative role of BMAL1 on *Il-6* expression in hypothalamic neurons. Additionally, palmitate did not alter *I*κ*b*α, *Il-6*, nor *Tlr4* in mHypoA-BMAL1-WT/F neurons unlike what was observed in mHypoE-46 cells. As the mHypoA-BMAL1-WT/F cell line is heterogeneous compared to the clonal mHypoE-46 cell line, it is possible that not all neuronal subpopulations within the mHypoA-BMAL1-WT/F cell line have the same response to palmitate. For example, the mHypoA-BMAL1-WT/F cell line contains POMC-expressing neurons, which did not have robust inflammatory responses to palmitate at 24 h as *Il-6* was not induced (unpublished observation, DDB).

Besides acting as an intracellular enzyme in the NAD+ salvage pathway, NAMPT can also be secreted as a cytokine to extracellular space. Higher levels of extracellular NAMPT, also known as visfatin, are associated with metabolic disorders. A meta-analysis uncovered a positive correlation between circulating NAMPT and parameters of obesity (60). Furthermore, several studies reported considerably higher plasma levels of circulating NAMPT in T2DM patients than in healthy controls, independent of BMI (61–63). Whether NAMPT is secreted from hypothalamic neurons is still unclear. In the CNS, glia but not neurons, may secrete NAMPT as it was observed in primary glial cultures, but not primary mouse cerebral cortex neuronal cultures (64). Regardless, NAMPT has been demonstrated to play an important role in inflammation. TLR4 was recently demonstrated to be a receptor of secreted NAMPT, leading to the induction of NF-κB-mediated inflammation (65). NAMPT-mediated inflammation has been demonstrated in the periphery (65–67). Moreover, NAMPT was also demonstrated to induce inflammatory responses in microglia and the hypothalamus (68, 69). Thus, the inflammatory role of intracellular NAMPT shown herein, or of the secreted form, may dysregulate hypothalamic function.

In this study, we sought to elucidate the complex interplay between circadian dysregulation and neuroinflammation in the context of hypothalamic exposure to palmitate. Our study revealed that palmitate altered mRNA expression levels of *Nampt*, the circadian clock genes *Bmal1* and *Per2*, as well as the inflammatory genes *I*κ*b*α, *Nf-*κ*b, Il-6* and *Tlr4*, in hypothalamic neurons. The changes in *Nampt* expression did not require BMAL1 and conversely, changes in *Bmal1* and *Per2* did not require NAMPT; these findings suggest that these two systems do not interact at the mRNA level. We used the NAMPT inhibitor FK866 and a BMAL-1 KO cell line to demonstrate that both NAMPT and BMAL1 were involved in regulating inflammatory gene expression. Furthermore, NAMPT inhibition was able to attenuate palmitate-induced expression of inflammatory genes *Il-6* and *Tlr4*. Taken together, our findings demonstrate that hypothalamic neuroinflammation in response to palmitate involves the circadian clock and metabolism, and that NAMPT inhibition may have therapeutic effects for palmitate-induced hypothalamic neuroinflammation.

## Data Availability Statement

The datasets generated for this study are available on request to the corresponding author.

## Ethics Statement

All animal procedures were approved by and performed in accordance with the Animal Use Protocols at the University of Toronto.

## Author Contributions

AT, WH, NJ, and JC performed the experiments and performed the statistical analysis. AT, WH, NJ, JC, and DB contributed conception and design of the study. AT and WH organized the database. AT, WH, and JC wrote the first draft of the manuscript. DB edited and wrote sections of the manuscript. All authors contributed to manuscript revision, read and approved the submitted version.

## Conflict of Interest

The authors declare that the research was conducted in the absence of any commercial or financial relationships that could be construed as a potential conflict of interest.
